# Introduction to Physical Measurements

**Published:** 1874-07

**Authors:** 


*An Introduction to Physical Measurements, with Appendices on Absolute Electrical Measurements, etc.* By Dr. F. Kohlrausch. Translated from the second German Edition. By Thomas Hutchinson Walker, B. A., B. Sc., and Henry Richardson Proctor, F. C. S. New York: D. Appleton & Co., 1874. Buffalo: Martin Taylor.

There are but few points in this book which will be appreciated by the medical profession. It is a work evincing a large amount of study and application upon the part of its author and in many of the departments of science is of incalculable value to the student in assisting him to prove the correctnees of his formulas.

There is but little of it which is of value to the medical profession and that is wholly comprised in the pages devoted to Electrical Measurements. We doubt however if any physician will ever put the formulas to a practical test in the therapeutical application of Electricity.

